# Lymphocyte-to-Monocyte Ratio Might Serve as a Prognostic Marker in Young Patients with Tongue Squamous Cell Carcinoma

**DOI:** 10.3390/jpm14020159

**Published:** 2024-01-30

**Authors:** Sam Augustine Kandathil, Ina Peter Truta, Lorenz Kadletz-Wanke, Gregor Heiduschka, Stefan Stoiber, Lukas Kenner, Harald Herrmann, Harun Huskic, Faris F. Brkic

**Affiliations:** 1 Department of Otorhinolaryngology and Head and Neck Surgery, Medical University of Vienna, 1090 Vienna, Austria; sam.kandathil@meduniwien.ac.at (S.A.K.); n11710989@students.meduniwien.ac.at (I.P.T.); lorenz.kadletz-wanke@meduniwien.ac.at (L.K.-W.); gregor.heiduschka@meduniwien.ac.at (G.H.); 2 Division of Anatomy, Center for Anatomy and Cell Biology, Medical University of Vienna, 1090 Vienna, Austria; 3 Department of Pathology, Medical University of Vienna, 1090 Vienna, Austria; stefan.stoiber@meduniwien.ac.at (S.S.); lukas.kenner@meduniwien.ac.at (L.K.); 4 Christian Doppler Laboratory for Applied Metabolomics, 1090 Vienna, Austria; 5 Center for Biomarker Research in Medicine, 8010 Graz, Austria; 6 Unit for Pathology of Laboratory Animals, University of Veterinary Medicine Vienna, 1210 Vienna, Austria; 7 Department of Radiation Oncology, Medical University of Vienna, 1090 Vienna, Austria; harald.herrmann@meduniwien.ac.at (H.H.); harun.huskic@meduniwien.ac.at (H.H.)

**Keywords:** tongue squamous cell carcinoma, lymphocyte-to-monocyte ratio, prognostic markers

## Abstract

Background: Young patients with tongue squamous cell carcinoma (TSCC) mostly lack typical prognostic markers and face a dire prognosis. The aim of this study was to analyze the prognostic relevance of lymphocyte-to-monocyte ratio (LMR) in TSCC patients, with a special emphasis on patients under 45 years. Methods: This retrospective study included all patients primarily treated for TSCC. The prognostic relevance of LMR was investigated in terms of predicting the overallsurvival (OS) and disease-free survival (DFS). Results: A total of 74 patients were included and the young cohort (<45 years) comprised 27 individuals. The mortality and recurrence rates were 39.2% (*n* = 29) and 37.8% (*n* = 28), respectively. OS and DFS were significantly shorter in the low LMR group within the whole cohort. Furthermore, low LMR was associated with worse prognosis, particularly inferior OS (median OS 1.7 vs. 14.6 years, *p* = 0.0156) and worse DFS (median DFS 0.8 years vs. not reached, *p* = 0.0405) in the young patient cohort. Conclusions: Our results reveal that pretreatment LMR might become a prognostic tool for young TSCC patients, especially due to its availability. However, further studies on larger cohorts are necessary to validate our results.

## 1. Introduction

Head and neck squamous cell carcinomas (HNSCCs) are composed of tumors developing from malignant transformation of the mucosal epithelium of the oral cavity, pharynx, and larynx [1]. Head and neck cancer is the sixth most common malignancy worldwide, accounting for around 500,000 deaths annually [2]. Known anatomical sites for HNSCC development of HNSCC within the oral cavity are lips, buccal mucosa, hard palate, anterior tongue, floor of mouth, and retromolar trigone [1]. In particular, tongue squamous cell carcinoma (TSCC) presents a subgroup of oral cancers with persisting high incidence rates and poor survival rates [3,4] in spite of recent advances in therapeutic options [5,6,7,8,9,10,11,12]. Parallel to the rising incidence of human papillomavirus-positive (HPV^+^) HNSCC affecting mostly the oropharynx, typical risk factors for oral cavity HNSCC are alcohol consumption and tobacco smoking and especially the combination of both, resulting in synergistic effects driving carcinogenesis [13,14]. Another risk factor, primarily affecting the population in south (east) Asia, is betel nut chewing, and TSCC is the leading cause of cancer death in India among men [2,15]. In general, high incidence rates of oral cavity cancers are induced by extensive exposure to and consumption of carcinogens [13]. Due to the lack of effective screening strategies, clinical examination remains the most reliable method for early detection and diagnosis. TSCCs are primarily surgically resected, and radiation therapy presents an alternative single-modality therapy. In advanced disease, systemic therapy is the current standard of care [11]. Locoregional recurrence of disease is common; therefore, long-term follow-up is essential. Unfortunately, similar to other localizations of HNSCC, a significant portion of patients present with a late-stage cancer without any clinical history of premalignancy, leading to late diagnosis and corresponding poor survival rates [1,16].

The escalating incidence rate of TSCC is a cause for concern, particularly among individuals below the age of 45, a demographic that traditionally exhibits lower exposure to conventional risk factors like alcohol or tobacco consumption [17,18]. This rising trend poses a unique challenge as the molecular profiles of tumors in younger patients often differ from those in older individuals, potentially due to their limited exposure to carcinogens [19].

Regrettably, the prognosis for the younger TSCC population is often bleak, marked by high recurrence rates [20,21]. This underscores the critical need for easily available prognostic markers that can facilitate early detection and more effective risk stratification. Identifying such markers holds the key to enhancing the overall management and treatment outcomes for this vulnerable population. The urgency of this situation highlights the importance of ongoing research efforts to uncover novel diagnostic tools and therapeutic strategies tailored to the specific characteristics of TSCC in younger individuals. By developing a deeper understanding of the unique molecular signatures and biological pathways associated with this demographic, we can pave the way for targeted interventions that may ultimately improve the prognosis and quality of life for those affected by this concerning trend.

The significant role of the immune system in cancer is not only described in the hallmarks of cancer [22] but has been acknowledged by the successful introduction and FDA-approval of immune checkpoint inhibitors, such as Pembrolizumab and Nivolumab, into the therapeutic scheme of recurrent or metastatic HNSCC [11]. Furthermore, Durvalumab has been investigated in combination with Tremelimumab or alone versus standard of care [23]. Cancer deregulates the immune cells within the tumor microenvironment and systemically, with first deregulations being observed one year prior to diagnosis [24]. To quantify and correlate such deregulations of the immune system with the prognosis and survival of cancer patients, several immune-related markers based on blood cell counts have been described, such as neutrophil-to-lymphocyte ratio (NLR), platelet-to-lymphocyte ratio (PLR), and lymphocyte-to-monocyte ratio (LMR) [25,26,27,28]. The predictive value of LMR has been reported in different malignancies [29,30,31,32,33,34,35,36,37,38]. In particular, two studies [20,21] have investigated the role of LMR in HNSCC and showed promising results.

To address this critical gap in the literature, we conducted a study to assess the prognostic value of LMR specifically within the context of young TSCC patients. Despite the well-established significance of LMR in various medical conditions, its relevance in predicting outcomes for young individuals with TSCC remains unexplored. Our primary objective is to assess LMR as an easily obtainable and reliable immune-related marker for prognostic evaluation.

This study will be conducted retrospectively, including a cohort of patients diagnosed with TSCC, with particular attention to those under the age of 45 years. By narrowing our focus to this age group, we aim to unravel potential age-specific nuances in the prognostic value of LMR, considering the distinct clinical characteristics observed in younger TSCC patients. The retrospective design allows us to leverage existing patient data, enabling a robust analysis of LMR’s prognostic relevance. This investigation holds the promise of not only validating LMR as a prognostic marker for young TSCC patients but also shedding light on its potential role in more personalized treatment and follow-up regimens.

The implications of the findings of this study extend beyond the research field, potentially reshaping actual clinical practices and patient care. The integration of LMR into prognostic assessments holds the promise of becoming a widely available tool for clinicians, particularly in the evaluation of survival outcomes among young patients with TSCC. Its inclusion could enable clinicians to engage in more personalized risk stratification. This, in turn, has the potential to enhance the overall management and outcomes for this vulnerable population. The integration of LMR into routine clinical decision-making processes may not only refine prognostication accuracy besides others but also guide clinicians in tailoring therapeutic strategies, ensuring a more personalized and effective approach for young TSCC patients. As the scientific community continues to explore the clinical utility of LMR in different tumor entities, we provide evidence of the prognostic value of LMR in young TSCC patients. If validated in larger cohorts and diverse clinical settings, LMR may emerge as a new prognostic marker within HNSCC.

## 2. Materials and Methods

This was a single-center, observational, retrospective cohort study. Patients with histologically confirmed TSCC treated at the Medical University of Vienna between 2002 and 2012 were included in the study. Exclusion criteria for the cohort were prior therapy, secondary primary carcinoma, and ongoing immunosuppressive treatment. Ultimately, a total of 74 patients were included in the study. Of these, 27 were under the age of 45 and were included in the young cohort. Baseline and outcome data were collected retrospectively from electronic patient review charts and included age at diagnosis, sex, tumor site (oral tongue only), TNM classification (8th edition AJCC), overall survival (OS), disease-free survival (DFS), treatment regimen, absolute lymphocyte, and monocyte count. Notably, pathological TNM staging was used in patients treated with primary surgery, and the clinical staging was mentioned in non-surgically treated cases. We calculated the LMR, which is defined as the ratio of absolute lymphocyte count divided by absolute monocyte count. We included laboratory data that had been collected routinely 0–14 days prior to therapy.

Statistical analysis and graphical presentation were performed with Prism (GraphPad Prism version 10.0.0 for Windows, GraphPad Software, Boston, MA, USA, www.graphpad.com; accessed 10 September 2023). As a non-normal distribution was shown in the Shapiro–Wilk test, we presented descriptive data using median and range values. Correspondingly, the median LMR (3.3) was used as a cut-off (<3.3 was considered low). The survival analysis and comparison between groups with low and high LMR were performed for the whole cohort, followed by an analysis solely within the young cohort. Log-rank tests were used to compare OS and DFS between groups for the whole cohort and the young cohort (<45 years).

## 3. Results

### 3.1. Descriptive Results

A total of 74 patients were included in the study and 27 (36.5%) were under 45 years of age. The median age of the whole cohort was calculated as 53.7 years (range 21.6–76.1 years). The majority of patients were males (*n* = 49; 66.2%). Most patients presented with an early-stage local disease at the time of initial diagnosis. In total, 25 patients (33.8%) were diagnosed with a T1 primary tumor. Moreover, the majority of patients in our cohort did not show any signs of lymph node metastases. In 41 patients (55.4%), no regional spread could be detected. Distant metastases could be found in 3 patients (4.1%) of our cohort at the initial workup. Most of the patients in our cohort were primarily treated with surgery and post-operative radio-(chemo)therapy (*n* = 52; 70.3%). Notably, free-flap reconstruction was performed in the majority of surgically treated T3 and T4 tumors. Here, we used either the radial forearm free flap or the anterolateral thigh flap. Elective neck dissection for levels I through III (for N0 cases) was performed for T3 and T4 tumors. Otherwise, in the case of N+, selective or modified-radical neck dissections were performed, irrespective of the T stage. A total of 14 patients (18.9%) were treated with primary radio-(chemo)therapy. Palliative systemic therapy was the only treatment option possible for 8 patients (10.8%). Detailed patient baseline demographics for the whole and the young cohort are summarized in Table 1. Furthermore, a total of 29 patients died of disease during the follow-up (39.2%) and 28 patients developed a recurrent disease (37.8%). A total of 46 patients (62.1%) died of natural causes in the whole cohort and 15 patients (55.6%) in the young group.

### 3.2. Prognostic Relevance of LMR

**Figure 1:**
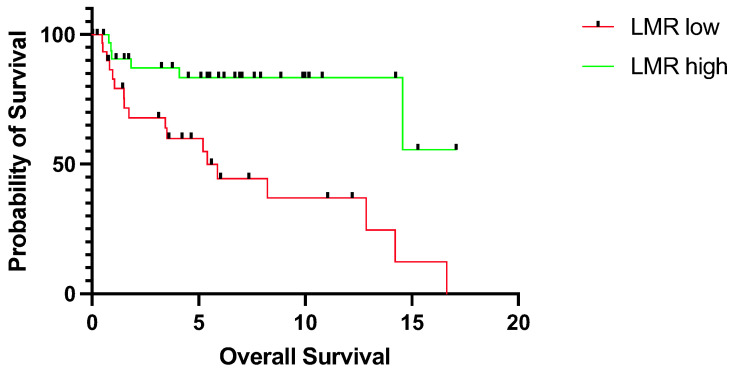
Overall survival for patients with tongue squamous cell carcinoma stratified into a low (*n* = 31) and high LMR group (*n* = 34) according to the median LMR value for overall survival. The survival times were statistically significantly different between groups (log-rank test *p* = 0.0007). Data of 9 patients were missing. LMR; lymphocyte-to-monocyte ratio.

**Figure 2:**
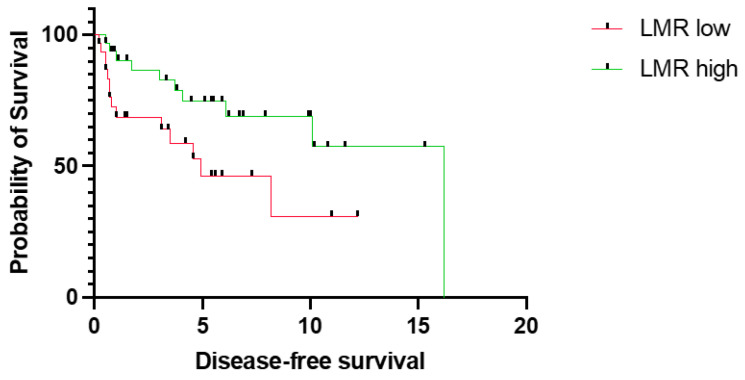
Disease-free survival for patients with tongue squamous cell carcinoma stratified into a low (*n* = 31) and high LMR group (*n* = 34). The survival times were statistically significantly different between patients with low and high LMR (log-rank test *p* = 0.0307). Data of 9 patients were missing. LMR; lymphocyte-to-monocyte ratio.

First, we sought to assess the prognostic relevance of LMR in our cohort. In particular, low LMR was significantly associated with worse OS (median OS 5.4 years vs. not reached, *p* = 0.0007, CI = 1.8–9.3 vs. 0.1–0.6) (Figure 1). Similarly, low LMR was significantly associated with shorter DFS (median DFS 4.9 years vs. 16.2 years, *p* = 0.0307, CI = 0.1–0.7 vs. 1.5–7.4) (Figure 2).

**Figure 3:**
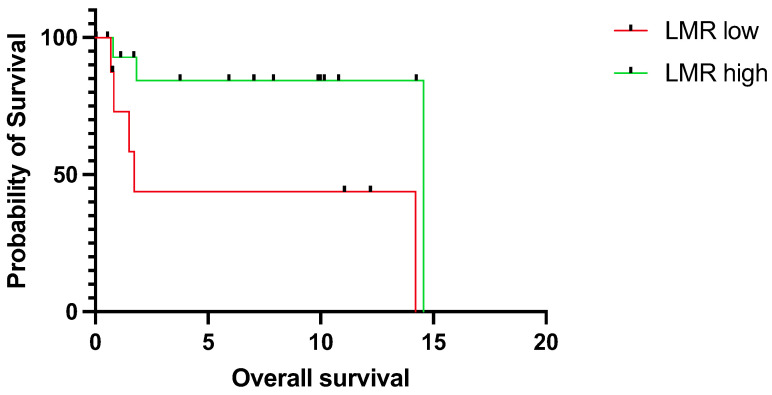
Overall survival for young patients (<45 years) with tongue squamous cell carcinoma stratified into a low (*n* = 8) and high LMR group (*n* = 16). The survival times were statistically significantly different between patients with low and high LMR (log-rank test *p* = 0.0156). Data of 3 patients were missing. LMR; lymphocyte-to-monocyte ratio.

**Figure 4:**
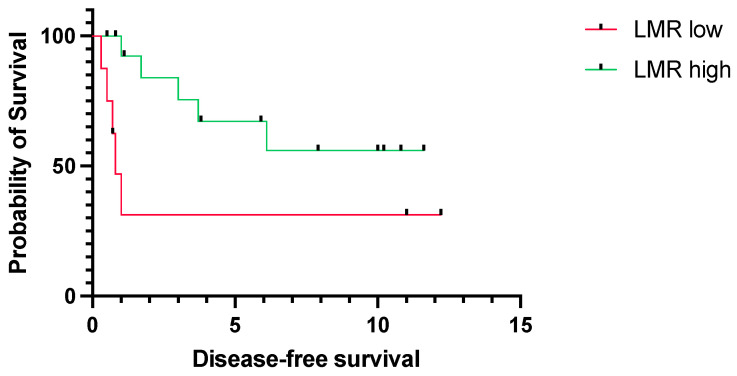
Disease-free survival for young patients (<45 years) with tongue squamous cell carcinoma stratified into a low (*n* = 8) and high LMR group (*n* = 16). The survival times were statistically significantly different between patients with low and high LMR (log-rank test *p* = 0.0405). Data of 3 patients were missing. LMR; lymphocyte-to-monocyte ratio.

Next, we assessed the predictive value of LMR, particularly for young patients. Correspondingly to the results in the whole cohort, low LMR was significantly associated with worse OS (median OS 1.7 years vs. 14.6, *p* = 0.0156, CI = 1.5–37.9 vs. 0.0–0.7) (Figure 3) and DFS (median DFS 0.8 years vs. not reached, *p* = 0.0405, CI = 1.1–21.33 vs. 0.0–0.9) (Figure 4).

## 4. Discussion

Our retrospective study provided evidence on the prognostic efficacy of pretreatment LMR in patients with TSCC. Given the tendency for dire survival rates among young individuals afflicted with this type of cancer, a need for easily obtainable prognostic markers is apparent for early risk stratification. These markers could potentially facilitate improved pre-treatment decisions and follow-up modalities. Notably, besides being significantly associated with worse OS and DFS in the whole TSCC cohort, low LMR was also shown to be significantly associated with shorter survival rates in a subgroup analysis within young patients (<45 years), who often face dire prognosis and recurrent disease [39].

Other research groups also aimed to investigate relevant prognostic factors in TSCC patients. Stationary markers, such as the protein expression of Signal Transducers and Activators of Transcription 3 (STAT3) and Cyclin D1, or whole cell populations within the tumor, particularly tumor-infiltrating leukocytes, were reported to be associated with survival outcomes [40]. Another factor that showed to be prognostically relevant in TSCC is tumor budding [41]. In particular, it was proven to be significantly associated with lymph node metastasis, DFS, and OS. However, these factors might be valuable in understanding the biological mechanisms behind better or worse survival but are not feasible for routine clinical monitoring. In contrast, circulating prognostic markers, which can be obtained in a rapid and cost-efficient manner, such as simple blood draws and subsequent analyses, are already implemented in routine work. 

Emerging evidence shows that chronic, local, and systemic inflammation is a driver of carcinogenesis and is an established hallmark of cancer [22]. Local inflammation serves as a catalyst for the constitutive activation of oncogenes and oncoproteins, including epithelial growth factor receptor (EGFR), STAT3, and Cyclin D1. These circumstances not only promote uncontrolled cell proliferation but also disrupt the physiological functions of non-cancerous cells within the tumor microenvironment, notably immune cells. The dysregulation of immune cells within the tumor microenvironment creates a microenvironment conducive to immune evasion, thereby undermining the body’s natural defense mechanisms [42]. On the other hand, the secretion of pro-inflammatory cytokines and growth hormones in the tumor microenvironment triggers systemic inflammation and tumor progression, which might influence the circulating immune cell count. Ratios based on these immune cell counts have been investigated for their prognostic relevance in various solid tumors [43,44,45] and have shown promising results in the past. The exploration of immune cell ratios as prognostic markers represents a significant advancement in cancer research, reflecting a shift toward understanding the systemic implications of cancer-related inflammation on tumor progression. These ratios, often derived from peripheral blood samples, hold promise as minimal-invasive tools for predicting disease outcomes [24].

HNSCC cells are notorious for their ability for immune evasion by promoting lower absolute lymphocyte cell counts, impaired natural killer cell activity, and poor antigen-presenting function [6]. Manipulating the interaction between tumor cells and immune cells by the use of immune checkpoint inhibitors has been one of the major breakthroughs in cancer therapy [46]. Checkpoint inhibitors targeting programmed death-1 (PD-1) Pembrolizumab and Nivolumab were approved for the treatment of recurrent and metastatic HNSCC. Additionally, Pembrolizumab was later approved for first-line treatment as monotherapy or combined with standard chemotherapy [47]. Another experimental checkpoint inhibitor targeting the cytotoxic T-Lymphocyte-associated protein 4 (CTLA4), Tremelimumab, was tested in combination with Durvalumab, a different PD-1 monoclonal antibody [23]. Notably, Durvalumab alone and in combination with the CTLA4 checkpoint inhibitor Tremelimumab was not superior to the standard of care.

Lymphocytes and their surface markers are indeed key players within the humoral and cellular antitumor response. Low lymphocyte levels dampen the immune response, and high monocyte levels promote local immune evasion of tumor cells and angiogenesis [48], which could partly explain the association of low LMR with worse survival. The prognostic relevance of LMR has already been investigated in head and neck malignancies. In particular, elevated LMR was associated with significantly improved OS and DFS in a study on 4260 HNSCC patients [49]. The association of poor prognosis and low LMR was also reported in other solid tumors, including bladder cancer [34], breast cancer [50], colorectal cancer [51], liver cancer [52], lung cancer [53], ovarian cancer [54], pancreatic cancer [55], prostate cancer [56], and thyroid cancer [57]. Finally, two studies [20,21] reported on the prognostic role of LMR in oral cancer. In both of these studies, low LMR was reported to be associated with poor OS. Furukawa et al. also highlighted that analyses of immunohistochemical stainings of tumor-infiltrating CD8^+^ (lymphocytes) and CD14^+^ (monocytes) cells were conducted, and a “CD8^+^/CD14^+^” ratio was calculated. High CD14^+^ cell counts were associated with poor OS [21]. Ong et al. suggest based on their results that patients with low pretreatment LMR should undergo close follow-up even after radical resection with a clear margin [20]. Importantly, to the best of our knowledge, our study was the first one focusing on the relevance of LMR in young TSCC patients, which urgently warrant easily obtainable predictive markers due to their aforementioned rising incidence rates and poor prognosis. Notably, our results correspond to results reported for other solid tumors and with previous studies within HNSCC depicting an association of low LMR with poor survival rates.

LMR is an easily obtainable marker, as absolute lymphocyte counts and monocyte counts can be evaluated at the beginning of the therapy through routine blood analyses and can potentially be continuously monitored throughout the therapy regimen and follow-up. Given that these counts are integral components of standard blood reports, the integration of LMR into routine clinical assessments is not only feasible but also practical. The incorporation of LMR as part of routine blood analyses adds a layer of convenience, making it a potentially valuable tool for clinicians in assessing and predicting treatment responses in HNSCC patients. Thus, following this retrospective analysis, future studies should be encouraged to analyze eventual dynamic changes of LMR in a prospective manner to further evaluate the strength of this prognostic marker. Moreover, a subgroup analysis focusing on human HPV^+^ HNSCC patients would add complexity, given the fact that HPV^+^ tumors typically exhibit a higher count of tumor-infiltrating leukocytes compared to HPV-negative (HPV^−^) HNSCC tumors [58]. Comparing LMRs in the context of HPV status may demask unique patterns and associations, potentially providing tailored prognostic insights for this distinct subset of HNSCC patients. 

The analysis of such environments could be interesting for investigation in various cell culture models [59]. The co-cultivation of HNSCC patient-derived organoids with patient’s own lymphocytes and monocytes and comparing predefined LMR groups (low vs. high LMR) could enable basic researchers for subsequent analysis in order to explore the biological mechanisms underlying the association between LMR and prognosis in HNSCC. Organoids have high genetic and phenotypic similarity to native tissue, maintaining the original intratumoral heterogeneity [60]. For instance, protein and gene expression analysis of organoids in low and high LMR environments could facilitate understanding of pro-tumor and anti-tumor activities within HNSCC. However, to mimic the function of a circulatory system, introducing perfusion systems such as organoid-on-chip systems would allow continuous perfusion and removal of waste from cells [61,62]. Another method would be to analyze tumor slices of tumor tissue in different co-culturing settings involving immune cells for further investigation of tumor and immune cell interaction [63,64]. Altogether, there are various modalities to further analyze the biological mechanisms of LMR without losing the clinical context by involving patient material within cell culture [59]. 

Another well-established prognosticator is the HPV positivity, indicating a better response to radiotherapy and therefore more favorable outcomes. However, it is only limited to oropharyngeal cancer. On the other hand, vast majority of other HNSCC, and particularly young TSCC patients, are HPV negative. This further underlines the need for novel prognostic markers for this patient group [65]. 

Moreover, drug resistance and radioresistance are still challenges in the management of HNSCC [16,66]. The interplay between immune cells, specifically lymphocytes and monocytes, has emerged as a critical factor, affecting initiation, progression, and spread of HNSCC and determining the response to immunotherapy [67]. The balance between lymphocytes and monocytes, as reflected by LMR, may be implicated in the modulation of chemo- and radioresistance in HNSCC.

A potential association between a low LMR and resistance to chemotherapy in HNSCC would suggest an immunological cause of treatment failure. Lymphocytes, particularly T cells, play a crucial role in recognizing and eliminating cancer cells [68]. However, the tumor microenvironment’s immunosuppressive characteristics may compromise the effectiveness of these immune responses, due to reduced T-cell infiltration, impaired T cell function, or upregulation of immune checkpoint molecules that hinder T-cell activity [69]. Consequently, tumors with a low LMR may create an immunosuppressive environment that protects cancer cells from the cytotoxic effects of chemotherapy by elevating stress-related pathways, such as autophagy and therefore leading to a poor treatment response [70]. Unraveling the interactions of lymphocytes with chemotherapy and understanding the impact of a low LMR on these interactions would be essential to overcome chemoresistance in HNSCC. Similarly, the role of lymphocytes and monocytes in modulating radioresistance in HNSCC warrants attention. Immune cells play a crucial role in amplifying the effects of radiotherapy through the bystander and abscopal effect and thus the stimulation of anti-tumor immune responses [71]. A low LMR may hamper these immune-mediated effects of radiotherapy. Additionally, monocytes and their derivatives, such as tumor-associated macrophages, can influence the response to radiotherapy. Depending on their polarization state, these cells can either enhance or suppress the effects of radiotherapy [72]. Altogether, strategies aimed at modulating the immune microenvironment to favor anti-tumor immune responses in HNSCC may enhance the effectiveness of radiotherapy and chemotherapy and mitigate the development of resistance. If the LMR might serve as a biomarker for therapy resistance, it is a matter for future studies.

Despite providing some novel findings, certain limitations must be acknowledged. First, the size of the investigated patient cohort was limited, especially with regard to patients under 45 years. Therefore, multivariate analysis was not possible and confounding factors (age, sex, stage, therapy regimen, etc.) cannot be excluded. Furthermore, the median LMR was used as the cut-off value. Thus, along with integral limitations of the retrospective study design (e.g., selection bias) of this work, validation of the cut-off in external larger cohorts data sets with a greater population of young TSCC patients is warranted.

## 5. Conclusions

Our study provided first evidence on the potential prognostic significance of LMR in young TSCC patients. Therefore, pretreatment LMR could potentially contribute to timely risk-stratification and enable proper adaptation of treatment and follow-up regimens. Indeed, our results warrant external validation, ideally in bigger patient cohorts.

## Figures and Tables

**Table 1:** Clinicopathological patient features stratified into young and whole cohorts. m, male; f, female; OS, overall survival; DFS, disease-free survival.

	Young Cohort (<45 years, *n* = 27)	Whole Cohort (*n* = 74)
Age		
Median, years (range)	34.2 (21.6–44.9)	53.7 (21.6–76.1)
Sex, n (%)		
m	18 (66.7)	49 (66.2)
f	9 (33.3)	25 (33.8)
T classification, n (%)		
T1	13 (48.1)	25 (33.8)
T2	8 (29.6)	36 (48.6)
T3	1 (3.7)	2 (2.7)
T4	5 (18.5)	11 (14.9)
N classification, n (%)		
N0	15 (55.6)	41 (55.4)
N1	4 (14.8)	17 (23.0)
N2	8 (29.6)	16 (21.6)
N3	0	0
M classification, n (%)		
M0	26 (96.3)	71 (95.9)
M1	1 (3.7)	3 (4.1)
Primary therapy, n (%)		
Surgery	20 (74.1)	52 (70.3)
Radio ± chemotherapy	6 (22.2)	14 (18.9)
Palliative systemic therapy	1 (3.7)	8 (10.8)
Clinical outcome, n (%)		
Median OS, years (range)	3.8 (0.0–14.6)	5.2 (0.0–17.1)
Median DFS, years (range)	1.7 (0.0–12.2)	3.5 (0.0–16.2)
Mortality	9 (33.3)	29 (39.2)
Recurrence	12 (44.4)	28 (37.8)

## Data Availability

The underlying data are available from the corresponding author upon a reasonable request.
